# Pathologic Nipple Discharge: Rare Imaging Presentation

**DOI:** 10.7759/cureus.23005

**Published:** 2022-03-09

**Authors:** Mariam Hanna

**Affiliations:** 1 Radiology, University of Florida College of Medicine, Gainesville, USA

**Keywords:** mri breast, breast mri, ductal carcinoma in-situ, nipple discharge, breast cancer management

## Abstract

A common presenting symptom for female patients is nipple discharge. Therefore having a background on how to manage is necessary for appropriately identifying and diagnosing the underlying etiology. The two most utilized imaging studies are diagnostic mammography and ultrasound. It can be difficult to identify a source/cause with mammography due to decreased sensitivity with also variable results seen with ultrasound. Advanced imaging such as MRI is becoming increasingly utilized limiting the need for ductography for diagnosis. In this case report, we discuss a rare case presentation of spontaneous nipple discharge.

## Introduction

Nipple discharge is the third most common breast-related symptom, following palpable mass and breast pain [1]. The main clinical objective is proper categorization of the discharge in order to determine appropriate management. Discharge can be classified into benign nipple discharge or pathologic nipple discharge [2]. Physiologic nipple discharge is characterized by bilateral multiple discharging ducts [2]. Pathologic discharge is characterized by unilateral or bloody discharge [3]. An intraductal papilloma is the most common cause of pathologic discharge followed by duct ectasia and malignancy as second and third common etiologies [3]. The reported incidence of malignancy is approximately 8% [3]. In this case, we discuss malignancy as the underlying pathology for patients’ nipple discharge.

## Case presentation

**Figure 1:**
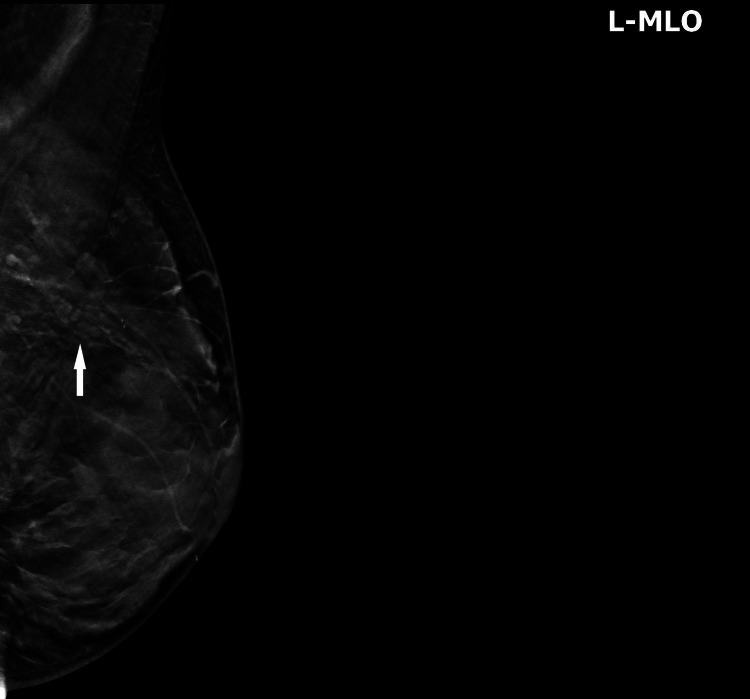
Left breast mediolateral oblique view digital image of the left breast demonstrating a focal asymmetry in the upper aspect of the left posteriorly (white arrow).

**Figure 2:**
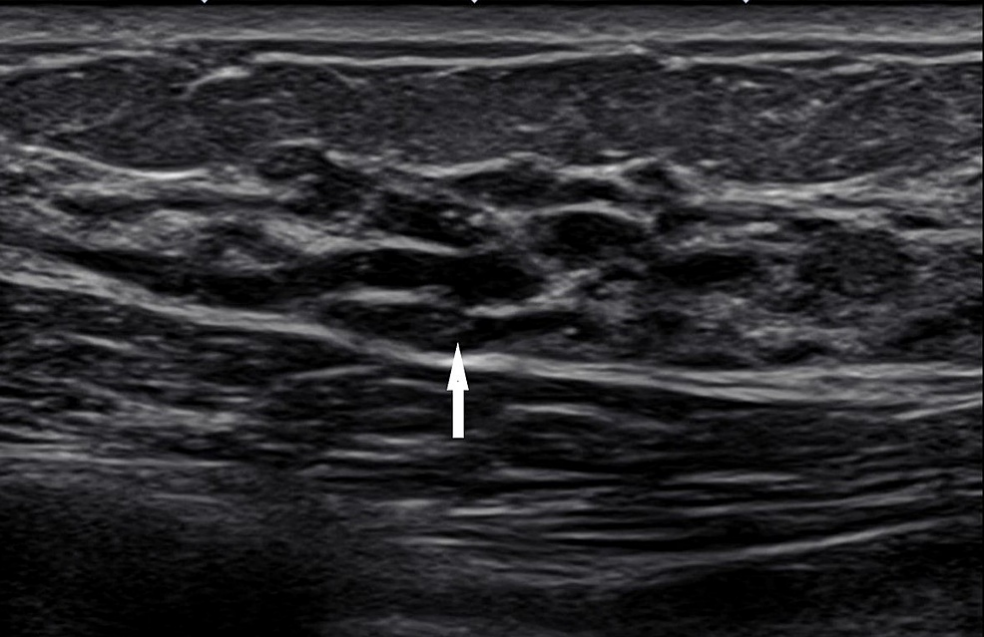
Grayscale transverse ultrasound image demonstrating dilated ducts/cystic appearance corresponding to the area of mammographic concern (white arrow).

**Figure 3:**
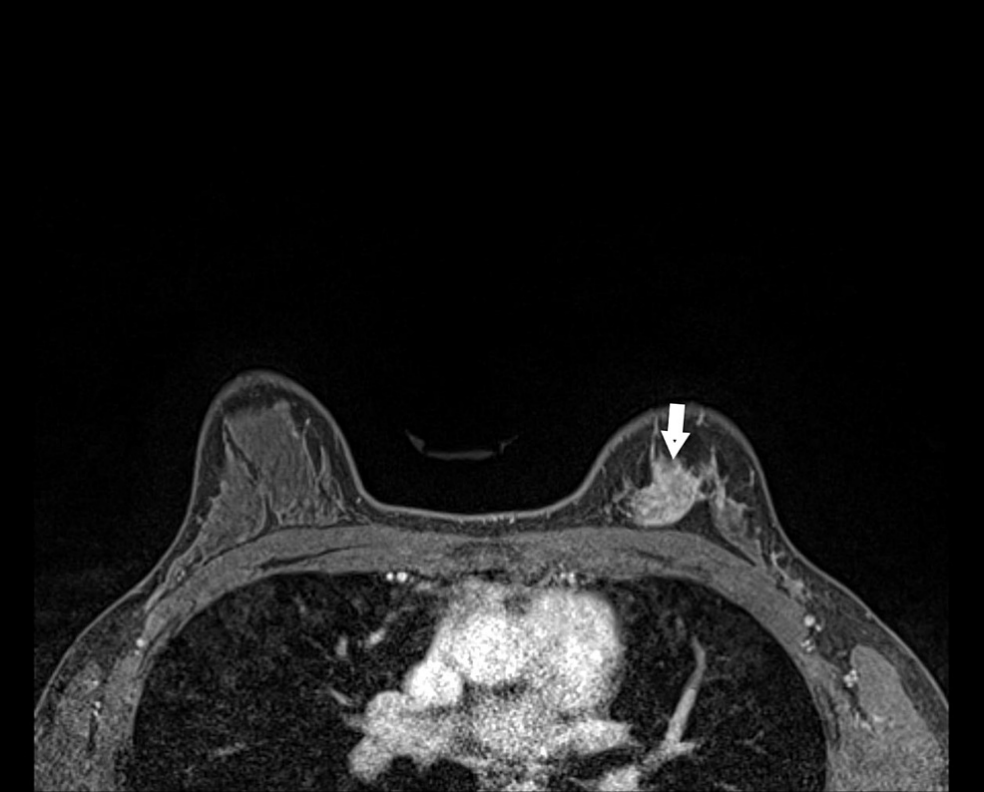
Single axial T1W post-contrast MRI image demonstrating enhancement in the upper inner quadrant of the left breast (white arrow).

The patient is a 41-year-old female presenting for evaluation of spontaneous left-sided bloody nipple discharge for several months. The patient noted that discharge has been increasing in quantity and frequency. Family history of breast cancer in aunt. No palpable mass was identified on physical exam. The patient underwent imaging evaluation with representative images displayed in Figures 1-3. It is important to include the clinical presentation along with imaging in order to reach the proper next step in management which is to biopsy the imaging abnormality. This case features an example of “pseudo microcystic” ductal carcinoma in situ (DCIS) caused by distention of the lobular portion of the terminal ductal lobular unit by DCIS (Figure 2) [1]. The sensitivities of breast MRI for detection of underlying cause of pathologic nipple discharge are 86% to 100% for invasive cancer and 40% to 100% for noninvasive disease and with a specificity of 97% (Figure 3) [1].

## Discussion

The mammographic and sonographic findings can often be nonspecific. The most common presenting feature is ductal dilatation [1]. Mammography is recommended as the initial imaging evaluation for nipple discharge. However, there has been concern that mammography can have low sensitivity for discharge evaluation [2]. An abnormality can present as a focal or diffuse finding. The mammographic presentation in this case is an asymmetry which has been documented in the literature to occur in approximately 2%-23% of cases on noncalcified DCIS (Figure 1) [2]. DCIS most commonly presents as calcifications mammographically and occurs in asymptomatic women during routine screening. This is the most common imaging manifestation on MRI for DCIS accounting for approximately 59% of all DCIS presentations [3]. Surgery has commonly been considered the gold standard for confirming the diagnosis and treating abnormal nipple discharge. Excision of the pathological ducts is needed to access the underlying cause. Nipple discharge can be a presenting symptom of early carcinomas [4-5]. All unilateral discharge needs to be assessed in order to exclude pathology [6-8]. It is important to raise breast cancer awareness and educate patients and providers with regards to atypical signs and symptoms as in this case.

## Conclusions

Pathologic nipple discharge can be a sign of breast cancer as illustrated in this case presentation. Careful history gathering and physical exam are extremely important in identifying the underlying etiology. Due to the wide spectrum of etiologies and differentials as potential causes varying from benign to invasive neoplasm, early detection is key. Both papillomas and neoplasms can have very similar imaging presentation when nipple discharge is the presenting symptom. Ductal carcinoma in situ has an overall good prognosis and when diagnosed early invasive neoplasms also have good outcomes. Imaging is very helpful and can serve as a powerful tool with breast MRI serving as a vital problem-solving examination. One should not stop investigating the underlying source of the pathologic discharge if imaging is negative but on the contrary, if no underlying abnormality is identified then the next step in management would be terminal duct excision since the presenting symptom is spontaneous nipple discharge.
